# Plasma inflammatory cytokines and treatment-resistant depression with comorbid pain: improvement by ketamine

**DOI:** 10.1186/s12974-021-02245-5

**Published:** 2021-09-15

**Authors:** Yanling Zhou, Chengyu Wang, Xiaofeng Lan, Hanqiu Li, Ziyuan Chao, Yuping Ning

**Affiliations:** 1 The Affiliated Brain Hospital of Guangzhou Medical University, Guangzhou Huiai Hospital, Mingxin Road #36, Liwan District, Guangzhou, 510370 China; 2 Guangdong Engineering Technology Research Center for Translational Medicine of Mental Disorders, Guangzhou, China; 3 The First School of Clinical Medicine, Southern Medical University, Guangzhou, China

**Keywords:** Ketamine, Treatment-resistant depression, Pain, Cytokine, IL-6

## Abstract

**Background:**

Treatment-resistant depression (TRD) and pain frequently coexist clinically. Ketamine has analgesic and antidepressant effects, but few studies have evaluated individual differences in antidepressant outcomes to repeated ketamine in TRD patients with comorbid pain. Our aims were to determine the difference in ketamine’s antidepressant effects in TRD patients with or without pain and then to examine whether inflammatory cytokines might contribute to ketamine’s effect.

**Methods:**

Sixty-six patients with TRD received six infusions of ketamine. Plasma levels of 19 inflammatory cytokines were assessed at baseline and post-infusion (day 13 and day 26) using the Luminex assay. Plasma inflammatory cytokines of sixty healthy controls (HCs) were also examined.

**Results:**

TRD patients with pain had a higher antidepressant response rate (*χ*^2^ = 4.062, *P* = 0.044) and remission rate (*χ*^2^ = 4.062, *P* = 0.044) than patients without pain. Before ketamine treatment, GM-CSF and IL-6 levels were higher in the pain group than in the non-pain and HC groups. In the pain group, levels of TNF-α and IL-6 at day 13 and GM-CSF, fractalkine, IFN-γ, IL-10, MIP-3α, IL-12P70, IL-17α, IL-1β, IL-2, IL-4, IL-23, IL-5, IL-6, IL-7, MIP-1β, and TNF-α at day 26 were lower than those at baseline; in the non-pain group, TNF-α levels at day 13 and day 26 were lower than those at baseline. In the pain group, the changes of IL-6 were associated with improvement in pain intensity (*β* = 0.333, *P* = 0.001) and depressive symptoms (*β* = 0.478, *P* = 0.005) at day 13. Path analysis showed the direct (*β* = 2.995, *P* = 0.028) and indirect (*β* = 0.867, *P* = 0.042) effects of changes of IL-6 on improvement in depressive symptoms both were statistically significant.

**Conclusion:**

This study suggested that an elevated inflammatory response plays a critical role in individual differences in TRD patients with or without pain. Ketamine showed great antidepressant and analgesic effects in TRD patients with pain, which may be related to its effects on modulating inflammation.

**Trial registration:**

ChiCTR, ChiCTR-OOC-17012239. Registered on 26 May 2017

**Supplementary Information:**

The online version contains supplementary material available at 10.1186/s12974-021-02245-5.

## Background

Depression and pain frequently coexist clinically [1–4]. The presence of comorbid depression and pain, compared to depression or pain alone, has been associated with greater treatment difficulties, poor functional outcomes, and increasing economic burdens on patients, families, and societies [5].

Although patients with comorbid depression and pain are common in clinics, the pathogenesis remains unclear. The mechanism of the interaction between depression and pain is likely more complex than that of a single disorder and suggests that the overlapping pathogenesis might have a role in the development of these two common disorders, including abnormal glutamate signaling, excessive activation of inflammation, and decreased brain-derived neurotrophic factor (BDNF) [6–8]. An abundance of evidence supports that chronic neuroinflammation independently plays a critical role in the pathophysiology of depression and pain and that alterations in inflammatory mediators have also been observed in this comorbid condition in recent years [9, 10]. Increased pro-inflammatory cytokine expression in the brain, especially tumor necrosis factor (TNF)-α, interleukin (IL)-6, and IL-1β in the hippocampus, amygdala, anterior cingulate, and frontal cortex, contributes to the development of depression pain comorbidity, which has been repeatedly shown in rodent models of neuropathic and inflammatory pain that also exhibit depressive-like behavior and, conversely, in rodent models of depression that also display altered nociceptive responses [11–13]. Imbalanced peripheral pro-inflammatory cytokine levels have also been independently observed in patients with depression and pain and in comorbid patients compared to healthy controls. For example, increased IL-6 levels can be found in patients with chronic pain and comorbid depressed mood, and IL-6 levels and depressive symptoms were positively associated with pain intensity [9]. TNF-α is another general inflammatory mediator reported to be increased in patients with depression and comorbid with pain, and augmented peripheral levels of TNF-α were associated with reduced pain thresholds in a correlative analysis [14]. Elevated peripheral C-reactive protein levels and depression symptoms were observed in patients with fibromyalgia or sciatica, while higher C-reactive protein levels correlated with greater depressive symptoms [15, 16]. Thus, taken together, these data suggest that a persistent inflammatory response may underlie comorbid depression and pain or at least partly contribute to the development of this comorbidity.

Currently, some analgesics and antidepressants are being used to treat this comorbidity; however, sometimes, there is limited clinical remission [17, 18]. Ketamine is an *N*-methyl-d-aspartic acid receptor antagonist with anesthetic and analgesic effects and has been used for acute pain for several decades. Recently, an increasing number of clinical studies have identified that a single subanesthetic dose of ketamine has a fast and robust antidepressant effect in patients with treatment-resistant depression (TRD) [19, 20]. Our previous results also showed greater effectiveness and longer remission periods with six infusions of intravenous ketamine for patients with TRD [21], consistent with other research [22]. Ketamine infusions also significantly reduced pain and depression in patients experiencing refractory neuropathic pain syndromes and depressive comorbidities [23, 24] and even successfully relieved depressive symptoms, suicidal ideation, and neuropathic pain in an adolescent with severe depression, suicidality, and neuropathic leg pain who failed multiple antidepressant and analgesic modalities [25]. In addition, oral ketamine thrice daily for 6 weeks was proven to have superior antidepressant effects compared to diclofenac for the treatment of depressed patients suffering from chronic pain [26].

Based on evidence from previous studies, ketamine may be ideal for the treatment of comorbid chronic pain and TRD; however, few clinical studies have evaluated individual differences in antidepressant outcomes to repeated ketamine infusions in TRD patients with pain. Moreover, although plasma levels of inflammatory cytokines decreased after six infusions of ketamine administration based on our previous results from a patient cohort with TRD and/or suicidal ideation [27], the roles of cytokines in ketamine’s effect on concurrent pain and depressive symptoms have not been explored. In the present study, we aimed to first determine the differences in ketamine’s antidepressant effects in TRD patients with and without the presence of painful symptoms and then to examine whether cytokines might contribute to ketamine’s effect in TRD patients with the presence of painful symptoms.

## Materials and methods

### Participants

We present a post hoc analysis of an original study designed to assess the antidepressant response of six adjunctive ketamine infusions in patients with TRD and/or suicidal ideation [21, 28]. This study was approved by the Clinical Research Ethics Committee of the Affiliated Brain Hospital of Guangzhou Medical University. All participants provided informed consent prior to participation.

This study included 66 patients with TRD who received six doses of ketamine, along with 60 healthy controls (HCs) matched with patients for age and sex. The main inclusion criteria for TRD patients were as follows: diagnosis of major depressive disorder (MDD) established using the Diagnostic and Statistical Manual of Mental Disorders (DSM-V) criteria, age between 18 and 65 years, 17-item Hamilton Depression Rating Scale (HAMD-17) score ≥ 17 at screening without hallucination or delusion, and treatment resistance defined as the failure of two adequate antidepressant trials. The exclusion criteria included the presence of alcohol or substance dependence or any serious or unstable medical conditions, including neurological, endocrine, rheumatic, and infective diseases. Taking anti-inflammatory agents (i.e., non-steroidal anti-inflammatory drugs or steroids) also was an exclusion criterion in the current analysis, but there was no participant taking anti-inflammatory agents in the original study. Current psychotropic medication had to be stable for ≥ 4 weeks, and the same dose was maintained during the six-infusion period. Additional detailed information regarding these participants has been described in our previous studies [21, 28, 29].

### Study design

TRD patients received ketamine three times weekly for 2 weeks. The detailed study design and methods have been previously published [21, 28, 29]. Intravenous ketamine (0.5 mg/kg) was administered over 40 min following an overnight fast via IV intravenous pump continuous infusion.

Depressive symptoms were assessed using the Montgomery-Asberg depression rating scale (MADRS) by clinicians at the pretreatment baseline, 24 h after each infusion, and again 14 days after the 6th infusion (day 26). A response was conventionally defined as a 50% or more reduction from baseline in MADRS score at 24 h after the 6th infusion (day 13). Remission was defined as a MADRS total score ≤ 10 at day 13.

Pain intensity was measured using the Short-Form McGill Pain Questionnaire (SF-MPQ). The main component of the SF-MPQ consists of the sensory index, affective index, present pain intensity (PPI) index, and visual analog scale (VAS). The VAS was assessed at the same time point as the MADRS, but the sensory index, affective index, and PPT were assessed at baseline, day 13, and day 26. Based on the presence or absence of pain using the SF-MPQ, 33 (50.0%) TRD patients had pain symptoms at baseline.

### Inflammatory cytokine measurements

TRD patients provided blood samples at baseline, day 13, and day 26, and HCs provided only a single blood sample. Blood samples were collected into EDTA tubes between 8:00 and 10:00 AM after an overnight fast. The tubes were immediately stored at + 4 °C and then centrifuged (3000 rpm/min at + 4 °C) for 10 min within 1 h. The plasma was obtained and stored at − 80 °C.

Plasma inflammatory cytokine levels were detected using the Human High Sensitivity T Cell Magnetic Bead Panel (Millipore, Billerica, MA, USA, HSTCMAG-28SK) performed with a Luminex 200 multiplex immunoassay system based on the manufacturer’s instructions. Nineteen cytokines, including interferon-inducible T cell alpha chemoattractant (ITAC), granulocyte-macrophage colony stimulating factor (GM-CSF), fractalkine, interferon (IFN)-γ, IL-10, macrophage inflammatory protein (MIP)-3a, IL-12P70, IL-13, IL-17A, IL-1β, IL-2, IL-4, IL-23, IL-5, IL-6, IL-7, IL-8, MIP-1β, and TNF-α, were quantified twice. The detailed detection process and interassay coefficients of variability have been described in our previous studies [27].

### Statistical analysis

Of the 66 TRD patients included, 5 (7.6%) lacked blood sample at day 26, three of them were in the non-pain group and two in the pain group. Thus, these data were analyzed based on the intent-to-treat with expectation-maximization algorithm interpolation method.

First, baseline demographic variables and clinical symptoms between the groups (pain vs. non-pain) were statistically evaluated using Student’s *t*-test for continuous variables with a normal distribution and the chi-square test for categorical variables.

Next, changes in MADRS scores, VAS, sensory index, affective index, and PPT over time and group differences were assessed using linear mixed models with group (pain vs. non-pain) and time (baseline, 24 h after each infusion or day 13, day 26) as factors. Baseline demographic and clinical variables that differed between the groups were entered as covariates. Bonferroni-corrected post hoc comparisons were used to calculate the group differences at each follow-up point. Cohen’s *d* was calculated to measure their difference.

Then, group comparisons of baseline cytokine levels among the pain group, non-pain group, and HC group were examined using multivariate analysis of covariance (MANCOVA), with age, gender, and body mass index as covariates. Post hoc analysis was used to compare the differences within each group. We further compared the changes of cytokine levels from baseline to day 13 and day 26 between the two patient groups using linear mixed models, with their baseline levels as covariates. Bonferroni-corrected post hoc comparisons were used to calculate the group differences at day 13 and day 26, as well as the differing levels between baseline and day 13 as well as day 26 within each group. Prior to the analyses, data for inflammatory cytokines were natural log-transformed.

Finally, linear regression analyses were used to further test whether the changes in depressive symptoms individually correlated with the changes in cytokine levels within each group and whether changes in pain symptoms correlated with the changes in inflammatory cytokine levels in the pain group. A mediation analysis using the process v2.15 in SPSS was used to investigate whether the relationships between changes in cytokine levels and changes in MADRS scores were mediated by the changes in VAS scores in the pain group.

All statistical analyses were performed using IBM SPSS Statistics version 22, and *P*-values < 0.05 were considered statistically significant. *P*-values were adjusted for multiple comparisons using false discovery rate correction.

## Results

### Demographics

At baseline, body mass index and years of education in the pain group were significantly greater than that in the non-pain group; other baseline demographic or clinical characteristics showed no statistically significant differences between the groups (Table 1).

**Table 1:** Demographics and clinical characteristics of ketamine in depressed patients with and without pain

Variables	Total (*n* = 66)	Non-pain (*n* = 33)	Pain (*n* = 33)	Statistics	*P*
Age, mean ± SD	35.8 ± 11.6	34.8 ± 11.5	36.8 ± 11.8	− 0.699	0.487
Male gender, *n* (%)	29 (43.9%)	13 (39.4%)	16 (48.5%)	0.554	0.457
Education (years), mean ± SD	12.2 ± 3.3	13.2 ± 2.9	11.1 ± 3.4	2.692	0.009
Employed, *n* (%)	31 (47.0%)	18 (54.5%)	13 (39.4%)	1.521	0.218
Body mass index (kg/m^2^), mean ± SD	22.7 ± 3.5	22.0 ± 3.6	23.4 ± 3.4	− 1.578	0.020
Duration of illness (months), mean ± SD	86.8 ± 76.6	77.9 ± 67.9	95.6 ± 84.5	− 0.989	0.326
Psychiatric comorbidity (yes), *n* (%)	11 (16.7%)	8 (24.2%)	3 (9.1%)	2.686	0.185
Family history of psychiatric disorders (positive), *n* (%)	20 (30.3%)	10 (30.3%)	10 (30.3%)	0.000	1.000
Dose of antidepressant (mg/day), mean ± SD	40.0 ± 21.4	36.8 ± 21.4	43.4 ± 21.2	− 1.243	0.218
On antipsychotic, *n* (%)	39 (59.1%)	21 (63.6%)	18 (54.5%)	0.564	0.453
On mood stabilizers, *n* (%)	14 (21.2%)	8 (24.2%)	6 (18.2%)	0.363	0.547
On benzodiazepines, *n* (%)	27 (40.9%)	12 (46.4%)	15 (45.5%)	0.564	0.453
Baseline MADRS score, mean ± SD	31.6 ± 7.6	31.2 ± 8.8	32.1 ± 6.4	− 0.449	0.655
Response rate after six infusions, *n* (%)	40 (60.6%)	16 (48.5%)	24 (72.7%)	4.062	0.044
Remission rate after six infusions, *n* (%)	26 (39.4%)	9 (27.3%)	17 (51.5%)	4.062	0.044
Days to response, mean ± SD	10.3 ± 8.1	12.4 ± 8.6	8.2 ± 7.2	2.130	0.037
Days to remission, mean ± SD	13.9 ± 7.7	15.8 ± 7.5	12.0 ± 7.6	2.025	0.047
Baseline VAS score, mean ± SD	2.4 ± 2.8	–	4.8 ± 2.2	NA	NA
Baseline sensory index, mean ± SD	2.2 ± 3.5	–	4.5 ± 3.8	NA	NA
Baseline affective index, mean ± SD	2.2 ± 3.1	–	4.3 ± 3.1	NA	NA
Baseline PPI, mean ± SD	1.1 ± 1.4	–	2.3 ± 1.1	NA	NA

*Abbreviations*: *MADRS*, Montgomery-Asberg Depression Rating Scale; *VAS*, visual analog scale; *PPI*, present pain intensity, *NA*, not applicable

### Efficacy of ketamine treatment

MADRS scores at baseline did not differ between the pain and non-pain groups. The patients with pain showed a significantly shorter time to an antidepressant response (*t* = 2.130, *P* = 0.037) and to depression remission (*t* = 2.025, *P* = 0.047) and had a higher response rate (*χ*^2^ = 4.062, *P* = 0.044) and remission rate (*χ*^2^ = 4.062, *P* = 0.044) than the patients without pain (Table 1).

**Fig. 1:**
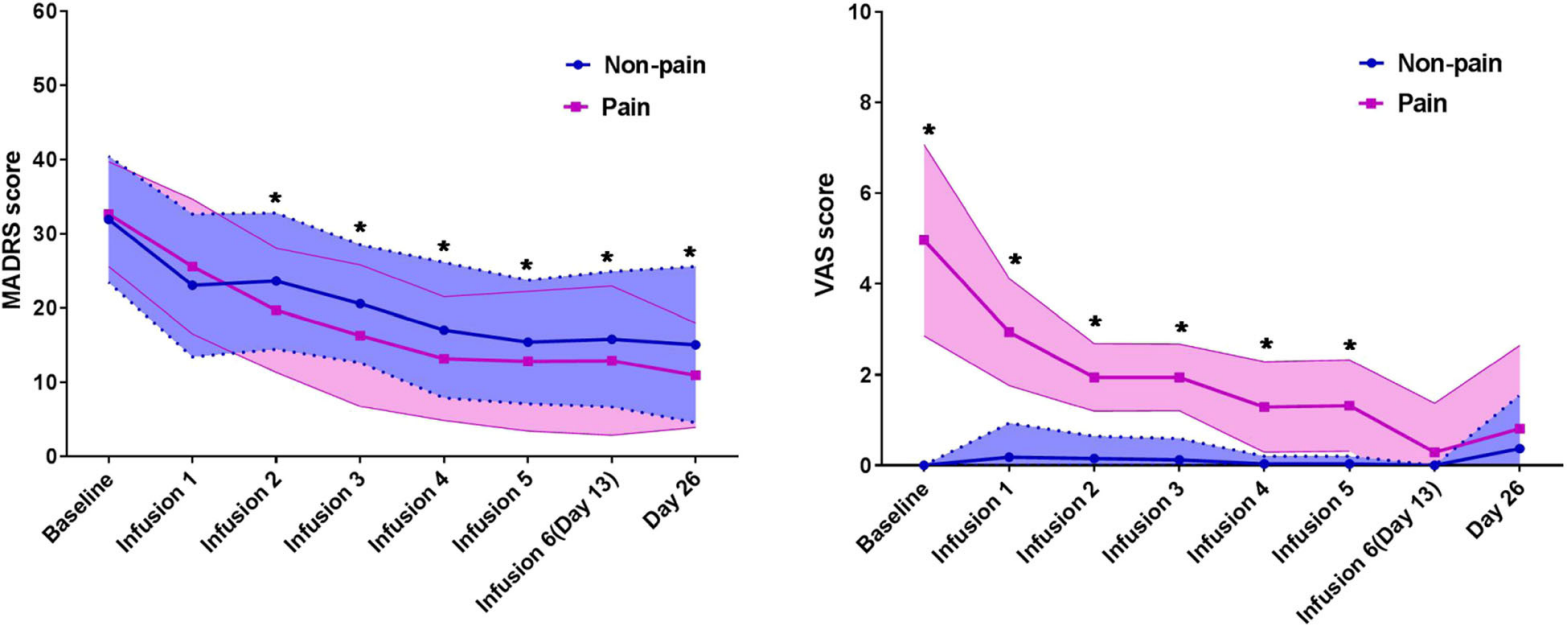
Change in depressive symptoms and pain intensity in the pain group and non-pain group. Legend: *significant difference at a given time point between the pain group and the non-pain group according to the post hoc analysis (*P* < 0.05). MADRS, Montgomery-Asberg Depression Rating Scale; VAS, visual analog scale

The linear mixed model with MADRS scores showed a significant group main effect (*F* = 12.669, *P* = 0.001), time main effect (*F* = 115.257, *P* < 0.001), and group-by-time interaction (*F* = 6.154, *P* = 0.001). Significant reductions in MADRS scores were found at 24 h after the first infusion compared to baseline scores, and these reductions were maintained over the subsequent infusion period as well as on day 26 in both groups (all *P* < 0.05, Fig. 1 and Supplementary Table 1). The pain group had lower MADRS scores than the non-pain group from the 2nd infusion to the 6th infusion and on day 26 (all *P* < 0.05). The largest significant differences in MADRS scores between the pain group and the non-pain group were observed at the 3rd infusion (Cohen’s *d* = 0.490).

**Fig. 2:**
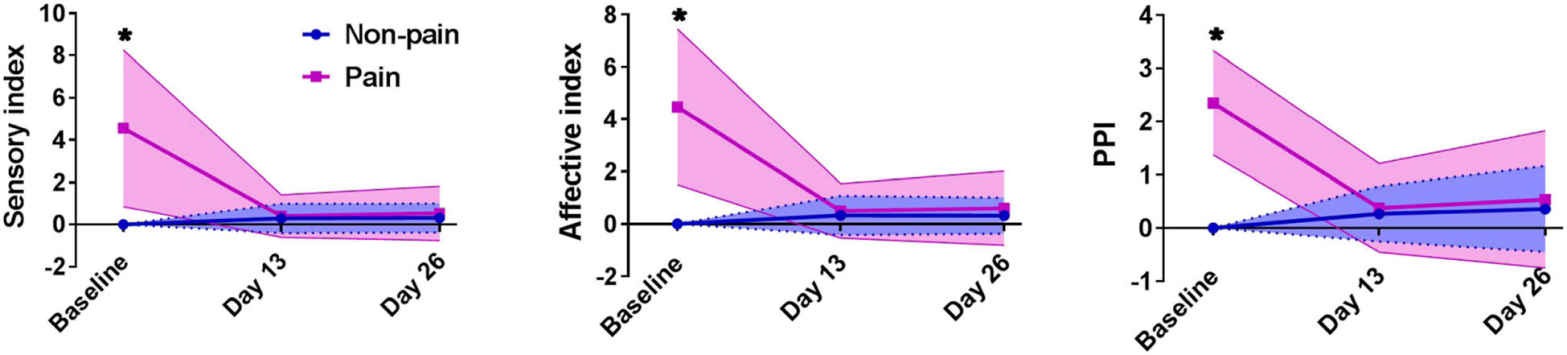
Change in the sensory index, affective index, and present pain intensity in the pain group and non-pain group. Legend: *significant difference at a given time point between the pain group and the non-pain group according to the post hoc analysis (*P* < 0.05). PPI, present pain intensity

The VAS, sensory index, affective index, and PPI scores at baseline are summarized in Table 1. The linear mixed model showed a significant group main effect, time main effect, group-by-time interaction with VAS (group: *F* = 488.157, *P* < 0.001; time: *F* = 80.631, *P* < 0.001; interaction: *F* = 92.561, *P* < 0.001), sensory index (group: *F* = 34.617, *P* < 0.001; time: *F* = 25.713, *P* < 0.001; interaction: *F* = 34.791, *P* < 0.001), affective index (group: *F* = 47.798, *P* < 0.001; time: *F* = 31.766, *P* < 0.001; interaction: *F* = 44.306, *P* < 0.001), and PPI (group: *F* = 48.350, *P* < 0.001; time: *F* = 20.567, *P* < 0.001; interaction: *F* = 38.981, *P* < 0.001). In the pain group, large and significant reductions in VAS scores were found at 24 h after the first infusion compared to baseline and were maintained over the subsequent infusion period as well as on day 26 (all *P* < 0.05, Fig. 1 and Supplementary Table 1), and significant reductions in the sensory index, affective index, and PPI were found at the 6th infusion and on day 26 compared to baseline (all *P* < 0.05, Fig. 2 and Supplementary Table 1). Significant differences between the pain group and the non-pain group in VAS scores were found during the preceding five infusions. No significant difference was found between the groups in the sensory index, affective index, or PPI at day 13 and day 26 (all *P* > 0.05).

### Differences in cytokine levels at baseline

**Fig. 3:**
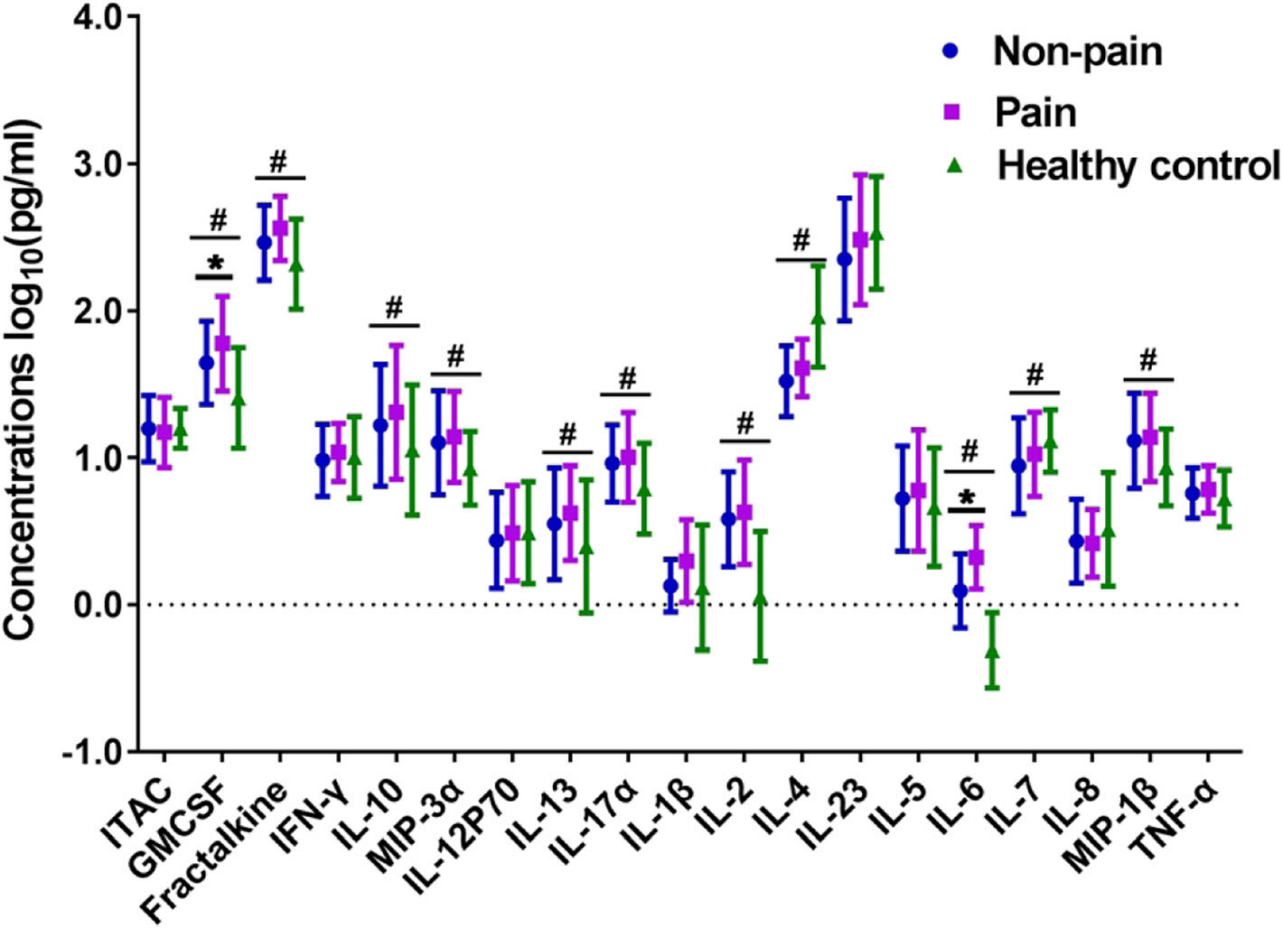
Baseline plasma levels of inflammatory cytokines in treatment-resistant depression patients with and without pain and healthy controls. Legend: ^#^significant difference among the pain group, non-pain group, and healthy controls (*P* < 0.05); *significant difference between the pain group and the non-pain group according to the post hoc analysis (*P* < 0.05). ITAC, interferon-inducible T cell alpha chemoattractant; GM-CSF, granulocyte-macrophage colony-stimulating factor; IFN, interferon; IL, interleukin; MIP, macrophage inflammatory protein; TNF, tumor necrosis factor

MANCOVA showed significant differences in the levels of 11 of the 19 measured cytokines, including GM-CSF, fractalkine, IL-10, MIP-3α, IL-13, IL-17α, IL-2, IL-4, IL-6, IL-7, and MIP-1β, among the pain, non-pain, and HC groups (*P* < 0.05). Post hoc analyses showed GM-CSF, fractalkine, IL-10, MIP-3α, IL-13, IL-17α, IL-2, IL-6, and MIP-1β levels were significantly higher in both the pain and non-pain groups than in the HC group, but IL-4 levels were significantly lower in both the pain and non-pain groups than in the HC group (*P* < 0.05); IL-7 levels were observed to be lowly expressed in the non-pain group as compared to the HC group; GM-CSF and IL-6 levels were significantly higher in the pain group than in the non-pain group (*P* < 0.05), while there was no significant difference in the other 17 inflammatory cytokines between the pain and non-pain groups (*P* > 0.05, Fig. 3 and Supplementary Table 2).

### Changes of cytokine after ketamine treatment

The linear mixed model showed significant time main effects on changes of GM-CSF, fractalkine, IFN-γ, IL-10, MIP-3α, IL-12P70, IL-17α, IL-1β, IL-2, IL-4, IL-23, IL-5, IL-6, IL-7, IL-8, MIP-1β, and TNF-α (*P* < 0.05) and significant group-by-time interaction on GM-CSF, fractalkine, IL-1β, and IL-6 (*P* < 0.05), while no significant group main effects on any of cytokines (*P* > 0.05, Supplementary Table 3). In the pain group, levels of TNF-α and IL-6 at day 13 and GM-CSF, fractalkine, IFN-γ, IL-10, MIP-3α, IL-12P70, IL-17α, IL-1β, IL-2, IL-4, IL-23, IL-5, IL-6, IL-7, MIP-1β, and TNF-α at day 26 were lower than those at baseline (*P* < 0.05); in the non-pain group, only TNF-α levels at day 13 and day 26 were lower than those at baseline (*P* < 0.05, Table 2).

**Table 2:** Comparison of inflammatory cytokine levels between post-infusion and baseline

Cytokines	Time	Non-pain	Compared with baseline		Pain	Compared with baseline	
		Mean ± SD	*t*	*P*	Mean ± SD	*t*	*P*
ITAC	Day 13	1.22 ± 0.25	− 0.857	1.000	1.22 ± 0.26	− 0.762	1.000
	Day 26	1.16 ± 0.24	0.690	1.000	1.16 ± 0.23	0.571	1.000
GMCSF	Day 13	1.68 ± 0.31	− 1.109	0.788	1.79 ± 0.38	0.891	1.000
	Day 26	1.62 ± 0.39	0.326	1.000	1.59 ± 0.37	5.109	< 0.001
Fractalkine	Day 13	2.52 ± 0.24	− 1.452	0.464	2.57 ± 0.26	0.476	1.000
	Day 26	2.44 ± 0.35	0.381	1.000	2.41 ± 0.28	4.381	< 0.001
IFN-γ	Day 13	1.00 ± 0.25	− 0.426	1.000	1.06 ± 0.26	− 0.085	1.000
	Day 26	0.96 ± 0.30	0.426	1.000	0.91 ± 0.26	3.710	0.005
IL-10	Day 13	1.22 ± 0.46	− 0.947	1.000	1.31 ± 0.51	0.456	1.000
	Day 26	1.08 ± 0.52	1.632	0.314	1.10 ± 0.54	4.053	< 0.001
MIP-3α	Day 13	1.12 ± 0.31	− 0.836	1.000	1.19 ± 0.27	0.091	1.000
	Day 26	1.04 ± 0.45	0.618	1.000	1.04 ± 0.35	2.964	0.011
IL-12P70	Day 13	0.46 ± 0.35	− 0.974	1.000	0.52 ± 0.33	0.051	1.000
	Day 26	0.39 ± 0.42	0.897	1.000	0.39 ± 0.37	3.385	0.003
IL-13	Day 13	0.52 ± 0.48	0.843	1.000	0.63 ± 0.32	− 0.098	1.000
	Day 26	0.52 ± 0.47	0.647	1.000	0.55 ± 0.48	1.588	0.338
IL-17α	Day 13	0.96 ± 0.30	− 0.333	1.000	1.06 ± 0.31	− 0.311	1.000
	Day 26	0.89 ± 0.42	1.267	0.615	0.88 ± 0.36	3.711	0.001
IL-1β	Day 13	0.17 ± 0.36	− 0.769	1.000	0.23 ± 0.39	1.635	0.313
	Day 26	0.09 ± 0.43	0.808	1.000	0.10 ± 0.39	4.288	< 0.001
IL-2	Day 13	0.59 ± 0.35	− 0.442	1.000	0.66 ± 0.40	0.186	1.000
	Day 26	0.49 ± 0.43	1.767	0.229	0.50 ± 0.40	0.395	< 0.001
IL-4	Day 13	1.46 ± 0.29	− 0.024	1.000	1.56 ± 0.25	0.098	1.000
	Day 26	1.44 ± 0.37	0.463	1.000	1.45 ± 0.24	2.951	0.012
IL-23	Day 13	2.37 ± 0.46	− 0.736	1.000	2.52 ± 0.45	0.264	1.000
	Day 26	2.23 ± 0.53	1.887	0.184	2.32 ± 0.50	3.981	< 0.001
IL-5	Day 13	0.74 ± 0.40	− 0.637	1.000	0.71 ± 0.41	2.218	0.087
	Day 26	0.66 ± 0.46	0.909	1.000	0.64 ± 0.41	3.473	0.002
IL-6	Day 13	0.19 ± 0.27	− 1.750	0.246	0.24 ± 0.19	1.850	0.043
	Day 26	0.14 ± 0.50	− 0.900	1.000	0.14 ± 0.36	3.600	0.001
IL-7	Day 13	0.97 ± 0.35	− 0.727	1.000	1.05 ± 0.32	0.114	1.000
	Day 26	0.88 ± 0.47	1.318	0.548	0.90 ± 0.32	3.705	0.001
IL-8	Day 13	0.49 ± 0.27	− 1.298	0.576	0.50 ± 0.28	− 1.170	0.738
	Day 26	0.38 ± 0.37	1.043	0.895	0.39 ± 0.30	1.170	0.738
MIP-1β	Day 13	1.07 ± 0.30	1.548	0.364	1.10 ± 0.34	0.452	1.000
	Day 26	1.07 ± 0.24	1.581	0.329	1.03 ± 0.36	2.774	0.016
TNF-α	Day 13	0.59 ± 0.17	4.341	< 0.001	0.67 ± 0.23	3.024	0.009
	Day 26	0.49 ± 0.25	6.585	< 0.001	0.52 ± 0.22	6.439	< 0.001

*Abbreviations*: *ITAC*, interferon-inducible T cell alpha chemoattractant; *GM-CSF*, granulocyte-macrophage colony-stimulating factor; *IFN*, interferon; *IL*, interleukin; *MIP*, macrophage inflammatory protein; *TNF*, tumor necrosis factor

### Clinical effects and inflammatory cytokine alterations after ketamine infusions

In the non-pain group, linear regression analyses showed that none of the cytokine alterations was associated with the reductions in MADRS scores following ketamine treatment at day 13 or day 26 (all *P* > 0.05). In the pain group, the changes of IL-6 levels were associated with the reductions in VAS (*β* = 0.333, *P* = 0.001) and MADRS (*β* = 0.478, *P* = 0.005) scores at day 13 and associated with the reductions in VAS scores at day 26 (*β* = 0.307, *P* = 0.015). Thus, the mediation model in the pain group was further explored at day 13. Path analysis showed the direct (*β* = 2.995, *P* = 0.028) and indirect (*β* = 0.867, *P* = 0.042) effects of the changes of IL-6 levels on reductions in MADRS scores both were statistically significant, indicating improvements in depressive symptom severity were partly dependent of the improvements in pain symptoms.

## Discussion

Three important findings of the present study are as follows: First, repeated subanesthetic doses of ketamine had significantly superior antidepressant effects in TRD patients with comorbid pain compared with patients without pain. Second, before ketamine treatment, TRD patients with comorbid pain had an elevated inflammatory response compared with patients without pain and healthy controls. Third, ketamine exerted greater effects on modulating inflammation in TRD patients with pain than in patients without pain.

### Subanesthetic ketamine can improve pain and depression comorbidities

The present study showed that 50% of TRD patients had comorbid pain, consistent with previous reports that the comorbidity rate of chronic pain and depression was approximately 40 to 60% [3, 4]. Our findings that TRD patients with or without pain showed similar severity of depressive symptoms suggested that painful symptoms were independent of the degree of depression, which was inconsistent with previous results that patients with MDD comorbid with chronic pain suffered from more severe depression [30, 31]. Note that the present study sample was treatment-resistant with a mixture of medicines as compared to other reports of MDD [30, 31]. Different objects the study chooses may result in a different conclusion that was at odds with the previous result.

Pain adversely affects the treatment response and prognosis of depression and vice versa. Patients with comorbid pain and depression were reported to experience a worse response to analgesic therapy than those without depressive symptoms [32]. Patients who had more severe pain symptoms prior to selective serotonin reuptake inhibitor treatment experienced poorer responses [30]. For TRD patients with pain in our study, the response rate to six infusions of ketamine was 72.7%, and the remission rate was 51.5%, which were significantly higher than patients without pain. Moreover, patients with comorbid pain also showed a significantly shorter time to achieve treatment response and remission. The better antidepressant outcomes in TRD patients comorbid with pain indicated that ketamine works on the brain through mechanisms different from the mechanisms of common antidepressants. Interestingly, the pain group also showed mild pain during ketamine treatment, even after ketamine treatment. A systematic review reported that headache is the most common acute side effect after ketamine treatment, especially in patients given intravenous ketamine [33]. In the present study, pain symptoms were reported during 76 (19.2%) infusions from among the 396 total infusions of ketamine. Although most of them reported that the pain resolved shortly after dose administration, their VAS score, sensory index, affective index, and PPI still reflected pain symptoms because these assessments covered a 24-h post-infusion period.

Ketamine showed analgesic effects in patients suffering from acute and chronic pain, as well as rapidly robust antidepressant effects in patients with TRD. Several clinical studies have supported that subanesthetic doses of ketamine may be ideal for the treatment of pain and depression comorbidities. Subanesthetic ketamine can reduce depressive symptoms in chronic pain patients, even in patients with refractory neuropathic pain syndromes [23, 24]. Daily oral ketamine for 6 weeks also effectively improved depressive symptoms in patients with chronic pain with mild-to-moderate depression [26]. Furthermore, in animal studies, ketamine has been reported to relieve pain-induced depression, which is independent of its antinociceptive effect. The foregoing results proved ketamine’s antidepressant and analgesic effects and gave rise to an interesting finding that TRD patients with pain achieved greater antidepressant outcomes than those without pain and took a shorter time to reach those outcomes.

### Ketamine’s effect on modulating inflammation

A wealth of evidence supports the hypothesis that excessive activation of inflammation contributes to the pathophysiology of the comorbidity of pain and depression. Microglial activation in the hippocampus and thalamus was found in patients suffering from chronic fatigue syndrome who exhibited pain and depression using positron emission tomography scans [34], and microglial activation and increased inflammatory cytokine expression were found in pain- and mood-related brain regions in rodent models of depression-pain comorbidity [13, 35]. Activation of the inflammatory response was also found in patients with comorbid depression and pain. For example, higher plasma IL-6 levels were found in patients with chronic back pain and comorbid depression and in patients with burning mouth syndrome and depressive symptoms than in healthy controls [9, 10]. The present study revealed that, compared with healthy controls, TRD patients with or without pain both had elevated levels of both pro-inflammatory (e.g., IL-17α, IL-2, and IL-6) and anti-inflammatory (e.g., IL-10, IL-13) cytokines, as well as other cytokines (e.g., GM-CSF, fractalkine, MIP-3α, and MIP-1β), while anti-inflammatory IL-4 levels decreased in both the pain and without pain groups. These changes may be associated with the interaction between the immune-inflammatory response system (IRS) and the compensatory immune-regulatory reflex system (CIRS). In MDD, the IRS overreacts, as reflected by the increased levels of pro-inflammatory cytokines, which consequently induce the CIRS. The CIRS, associated with the elevation of T helper type 2 and T regulatory activities, results in increased anti-inflammatory cytokine levels, which in turn protect against the excessive IRS and restore a balanced state of the immune system [36, 37]. Therefore, the simultaneous activation of IRS and CIRS leads to the elevation of both pro-inflammatory and anti-inflammatory cytokines in patients with MDD, which was supported by several studies [38, 39]. However, anti-inflammatory IL-4 levels did not elevate in TRD patients, and IL-7 levels were observed to be lowly expressed in the non-pain group as compared to the healthy controls in the present study, suggesting a more complicated mechanism is involved in TRD.

Furthermore, plasma levels of GM-CSF and IL-6 in TRD patients with pain were higher than those in patients without pain, reflecting that these patients are much more likely to suffer an elevated inflammatory response. A preclinical study also reported increased levels of the inflammatory cytokines IL-6, IL-1β, TNF-α, IL-4, and IL-10 in spared nerve ligation rats with a depression-like phenotype but not rats without a depression-like phenotype [40]. Thus, an excessive inflammatory response may contribute to individual differences in the risks for the comorbidity of pain and depression.

Then, we further analyzed whether alterations in inflammatory cytokines were related to individual differences in ketamine’s effects on comorbid TRD and pain. Results of the linear mixed model showed no significant group main effects on any of cytokines, while most of the 19 inflammatory cytokine levels decreased after six infusions of ketamine in TRD patients as reflected by their significant time main effects, consistent with our previous findings in depressed patients without controlling pain [27]. These alterations were observed in TRD patients with pain, including GM-CSF, fractalkine, IFN-γ, IL-10, MIP-3α, IL-12P70, IL-17α, IL-1β, IL-2, IL-4, IL-23, IL-5, IL-6, IL-7, MIP-1β, and TNF-α, while only TNF-α levels decreased after ketamine infusions in patients without pain. We speculate that there is a relationship between the modulation of inflammatory response and ketamine’s superior antidepressant effects in TRD patients with pain. Moreover, given that the TRD patients with pain exhibited higher plasma IL-6 and GM-CSF levels than the patients without pain before ketamine intervention, it is likely that patients who have elevated inflammatory responses may more easily benefit from ketamine. Similarly, higher IL-6 levels were reported as a potential predictor of ketamine’s antidepressant efficacy in a clinical study [41]. In an animal study, spared nerved ligation rats with a depression-like phenotype showed lower serum levels of IL-1β and IL-6 than non-responders at baseline [40]. Results from rats subjected to inescapable electric shock suggested that peripheral IL-6 may contribute to resilience versus susceptibility to inescapable stress [42]. In addition, it was important to note that most cytokines showed significant reduction at 2 weeks after six ketamine infusions compared with baseline, not at day 13, indicating a possible existence of a delayed response in peripheral inflammatory cytokines like that in the brain after ketamine infusions in TRD patients with pain. Previous studies demonstrated that the response of inflammatory cytokines was out-of-sync with depressive symptoms after antidepressant treatment [37, 43]. Moreover, the IRS and CIRS pathways were active even in the remission stage of a mood disorder, which suggested that the original steady state may not be restored immediately after an acute episode [38].

Notably, anti-inflammatory IL-10 and IL-4 levels also decreased 2 weeks after the final ketamine infusion, which were significantly elevated in the pain group before ketamine treatment compared with healthy controls. These changes in IL-10 and IL-4 levels after ketamine treatment may be a result of the weakening of the CIRS response caused by decreased IRS activity. Similarly, our previous study found a significant decreased in a broad range of cytokines, also including anti-inflammatory IL-10 and IL-4 after 4 weeks of antidepressant treatment in patients with first-episode drug-naive MDD [44]. A recent meta-analysis including 32 studies also revealed a significant decrease in both pro-inflammatory (e.g., IL-6) and anti-inflammatory (e.g., IL-4, IL-10) cytokine levels after antidepressant treatment [45].

Interestingly, correlations between changes in IL-6 levels and both antidepressant and analgesic effects were found in TRD patients with pain at day 13; however, further analysis showed that ketamine’s analgesic effect mediated the association between decreases of IL-6 levels and its antidepressant effect. Previous studies have also suggested that ketamine can decrease the expression of inflammatory cytokines in MDD patients, but the results regarding the relationship between the changes in cytokine levels and antidepressant efficacy have been inconsistent [46, 47]. Chen et al. found that the decrease in levels of TNF-α after a single dose of ketamine in patients with MDD was correlated with antidepressant efficacy [46], while no association was found in Park’s clinical study [47]. In combination with the present results, the modulation of inflammation, especially the decrease of IL-6 levels, may play a more direct role in ketamine’s analgesic effect than its antidepressant effect. However, the precise mechanisms underlying the relationship between elevated inflammatory responses and susceptibility to the comorbidity of pain and depression are currently unknown. Further preclinical studies are warranted to determine the precise anti-inflammatory mechanism of ketamine in combined models of TRD and pain.

### Limitations

This study was associated with several limitations. First, the small patient sample size meant that it is impossible to perform subgroup analyses by the exact area of pain. Second, seven participants lacked inflammatory cytokine data at 2 weeks after ketamine treatment. The third limitation was that inflammatory cytokines were measured only in the peripheral blood, which does not directly reflect the inflammatory response in the brain. Finally, the absence of cytokine data in healthy volunteers at days 13 and 26 meant that we could not know how the inflammatory signatures of patients compare to healthy volunteer values at these points.

## Conclusion

Our study suggested that an elevated inflammatory response plays a critical role in the individual differences among TRD patients with or without pain. Ketamine showed great antidepressant and analgesic effects in TRD patients with pain, which may be related to its effect on modulating inflammation. Further preclinical studies to address the precise modulating inflammation mechanism of ketamine and future therapies based on such a mechanistic understanding can be developed to better serve those with TRD and pain comorbidity.

## Supplementary Information

**Additional file 1: Table S1.** Change in depressive symptoms and pain intensity in pain group and non-pain group. Abbreviations: MADRS, Montgomery-Asberg Depression Rating Scale; VAS, Visual Analogue Scale; PPI, Present Pain Intensity. **Table S2.** Baseline plasma levels of inflammatory cytokines in pain group, non-pain group and healthy controls. Abbreviations: ITAC, interferon-inducible T cell alpha chemoattractant; GM-CSF, granulocyte macrophage colony-stimulating factor; IFN, interferon; IL, interleukin; MIP, macrophage inflammatory protein; TNF, tumor necrosis factor; HCs, healthy controls. **Table S3.** Comparison of inflammatory cytokines levels between groups using linear mixed model analysis. Abbreviations: ITAC, interferon-inducible T cell alpha chemoattractant; GM-CSF, granulocyte macrophage colony-stimulating factor; IFN, interferon; IL, interleukin; MIP, macrophage inflammatory protein; TNF, tumor necrosis factor.

## Data Availability

The datasets generated during and/or analyzed during the current study are not publicly available due to the intelligence rights owned by the hospital and the authors.

## Abbreviations

BDNF: Brain-derived neurotrophic factor
CIRS: Compensatory immune-regulatory reflex system
DSM-V: Diagnostic and Statistical Manual of Mental Disorders
GM-CSF: Granulocyte-macrophage colony-stimulating factor
HAMD-17: 17-Item Hamilton Depression Rating Scale
HC: Healthy control
IFN: Interferon
IL: Interleukin
IRS: Immune-inflammatory response system
ITAC: Interferon-inducible T cell alpha chemoattractant
MADRS: Montgomery-Asberg Depression Rating Scale
MDD: Major depressive disorder
MIP: Macrophage inflammatory protein
PPI: Present pain intensity
SF-MPQ: Short-Form McGill Pain Questionnaire
TNF: Tumor necrosis factor
TRD: Treatment-resistant depression
VAS: Visual analog scale
