# Supplementary material for: Plasma inflammatory cytokines and treatment-resistant depression with comorbid pain: improvement by ketamine
Source: J Neuroinflammation. 2021 Sep 15;18:200. doi: 10.1186/s12974-021-02245-5 (PMC8444441; doi:10.1186/s12974-021-02245-5)
Supplement: Supplementary file 1 — Additional file 1: Table S1. Change in depressive symptoms and pain intensity in pain group and non-pain group. Abbreviations: MADRS, Montgomery-Asberg Depression Rating Scale; VAS, Visual Analogue Scale; PPI, Present Pain Intensity. Table S2. Baseline plasma levels of inflammatory cytokines in pain group, non-pain group and healthy controls. Abbreviations: ITAC, interferon-inducible T cell alpha chemoattractant; GM-CSF, granulocyte macrophage colony-stimulating factor; IFN, interferon; IL, interleukin; MIP, macrophage inflammatory protein; TNF, tumor necrosis factor; HCs, healthy controls. Table S3. Comparison of inflammatory cytokines levels between groups using linear mixed model analysis. Abbreviations: ITAC, interferon-inducible T cell alpha chemoattractant; GM-CSF, granulocyte macrophage colony-stimulating factor; IFN, interferon; IL, interleukin; MIP, macrophage inflammatory protein; TNF, tumor necrosis factor. [file 12974_2021_2245_MOESM1_ESM.doc]

Supplemental table 1. Change in depressive symptoms and pain intensity in pain group and non-pain group.

| Variables | Time | Non-pain  (n=33) | Pain  (n=33) | t | *P* | Cohen’s d |
| --- | --- | --- | --- | --- | --- | --- |
| MADRS score | Infusion 1 | 23.06±9.61 | 25.59±9.06 | 0.435 | 0.663 | -0.271 |
|  | Infusion 2 | 23.65±9.17 | 19.71±8.36 | 3.433 | 0.001 | 0.449 |
|  | Infusion 3 | 20.59±7.92 | 16.29±9.54 | 3.724 | <0.001 | 0.490 |
|  | Infusion 4 | 17.03±9.11 | 13.21±8.34 | 3.478 | 0.001 | 0.438 |
|  | Infusion 5 | 15.44±8.32 | 12.85±9.41 | 3.506 | 0.001 | 0.291 |
|  | Infusion 6 (Day 13) | 15.82±9.31 | 12.91±10.05 | 4.184 | <0.001 | 0.303 |
|  | Day 26 | 15.07±10.53 | 10.97±7.03 | 3.029 | 0.003 | 0.458 |
| VAS score | Infusion 1 | 0.18±0.76 | 2.94±1.18 | -16.419 | <0.001 | -2.785 |
|  | Infusion 2 | 0.15±0.50 | 1.94±0.75 | -9.705 | <0.001 | -2.816 |
|  | Infusion 3 | 0.12±0.48 | 1.94±0.74 | -10.144 | <0.001 | -2.933 |
|  | Infusion 4 | 0.03±0.17 | 1.29±1.00 | -5.557 | <0.001 | -1.755 |
|  | Infusion 5 | 0.03±0.18 | 1.32±1.01 | -5.261 | <0.001 | -1.786 |
|  | Infusion 6 (Day 13) | 0.00±0.00 | 0.29±1.09 | -1.249 | 0.213 | -0.377 |
|  | Day 26 | 0.37±0.19 | 0.81±1.84 | -1.574 | 0.117 | -0.284 |
| Sensory index | Day 13 | 0.30±0.70 | 0.41±1.01 | -0.293 | 0.769 | -0.122 |
|  | Day 26 | 0.32±0.69 | 0.54±1.29 | -0.507 | 0.613 | -0.208 |
| Affective index | Day 13 | 0.33±0.76 | 0.50±1.05 | -0.423 | 0.673 | -0.182 |
|  | Day 26 | 0.32±0.69 | 0.61±1.42 | -0.693 | 0.489 | -0.257 |
| PPI | Day 13 | 0.27±0.52 | 0.38±0.83 | -0.512 | 0.610 | -0.158 |
|  | Day 26 | 0.36±0.81 | 0.54±1.29 | -0.768 | 0.445 | -0.167 |

Abbreviations: MADRS, Montgomery-Asberg Depression Rating Scale; VAS, Visual Analogue Scale; PPI, Present Pain Intensity.

Supplemental table 2. Baseline plasma levels of inflammatory cytokines in pain group, non-pain group and healthy controls.

| Variables | Non-pain  (n=33) | Pain  (n=33) | HCs  (n=60) | F | *P* | Ajusted *P* | Post hoc tests |
| --- | --- | --- | --- | --- | --- | --- | --- |
| ITAC | 1.21±0.23 | 1.23±0.25 | 1.20±0.14 | 0.224 | 0.800 | 15.200 |  |
| GMCSF | 1.60±0.29 | 1.85±0.31 | 1.43±0.30 | 15.189 | <0.001 | 0.000 | Pain > Non-pain > HCs |
| Fractalkine | 2.48±0.30 | 2.63±0.25 | 2.36±0.34 | 9.004 | <0.001 | 0.000 | Pain, Non-pain > HCs |
| IFN-γ | 1.03±0.26 | 1.05±0.21 | 1.06±0.33 | 0.398 | 0.672 | 4.256 |  |
| IL-10 | 1.22±0.45 | 1.30±0.52 | 1.05±0.43 | 3.843 | 0.029 | 0.046 | Pain, Non-pain > HCs |
| MIP-3α | 1.16±0.40 | 1.13±0.34 | 0.90±0.31 | 6.801 | 0.002 | 0.003 | Pain, Non-pain > HCs |
| IL-12P70 | 0.43±0.33 | 0.51±0.36 | 0.52±0.45 | 0.281 | 0.755 | 7.173 |  |
| IL-13 | 0.64±0.40 | 0.68±0.33 | 0.47±0.50 | 3.771 | 0.026 | 0.055 | Pain, Non-pain > HCs |
| IL-17α | 1.01±0.38 | 1.03±0.33 | 0.84±0.32 | 6.766 | 0.002 | 0.003 | Pain, Non-pain > HCs |
| IL-1β | 0.14±0.23 | 0.35±0.32 | 0.16±0.40 | 3.297 | 0.040 | 0.095 |  |
| IL-2 | 0.62±0.34 | 0.66±0.43 | 0.12±0.46 | 30.881 | <0.001 | 0.000 | Pain, Non-pain > HCs |
| IL-4 | 1.52±0.24 | 1.61±0.20 | 1.96±0.33 | 27.033 | <0.001 | 0.000 | Pain, Non-pain < HCs |
| IL-23 | 2.33±0.41 | 2.55±0.46 | 2.52±0.41 | 2.152 | 0.121 | 0.328 |  |
| IL-5 | 0.72±0.44 | 0.83±0.45 | 0.73±0.46 | 0.921 | 0.401 | 1.905 |  |
| IL-6 | 0.12±0.33 | 0.36±0.27 | -0.31±0.25 | 17.777 | <0.001 | 0.000 | Pain > Non-pain > HCs |
| IL-7 | 0.90±0.31 | 1.01±0.33 | 1.12±0.25 | 4.530 | 0.013 | 0.022 | Non-pain < HCs |
| IL-8 | 0.44±0.36 | 0.45±0.20 | 0.53±0.47 | 1.168 | 0.314 | 1.193 |  |
| MIP-1β | 1.10±0.37 | 1.19±0.32 | 0.90±0.34 | 7.111 | 0.001 | 0.001 | Pain, Non-pain > HCs |
| TNF-α | 0.83±0.23 | 0.85±0.24 | 0.78±0.21 | 1.354 | 0.262 | 0.830 |  |

Abbreviations: ITAC, interferon-inducible T cell alpha chemoattractant; GM-CSF, granulocyte macrophage colony-stimulating factor; IFN, interferon; IL, interleukin; MIP, macrophage inflammatory protein; TNF, tumor necrosis factor; HCs, healthy controls.

Supplemental table 3. Comparison of inflammatory cytokines levels between groups using linear mixed model analysis.

| Cytokines | Time effect | | Group effect | | Time by group interaction | |
| --- | --- | --- | --- | --- | --- | --- |
|  | F | P | F | P | F | P |
| ITAC | 2.126 | 0.123 | 0.000 | 0.991 | 0.013 | 0.988 |
| GMCSF | 9.611 | <0.001 | 1.158 | 0.286 | 4.382 | 0.009 |
| Fractalkine | 13.431 | <0.001 | 0.529 | 0.470 | 3.234 | 0.043 |
| IFN-γ | 5.058 | 0.008 | 2.252 | 0.109 | 0.547 | 0.462 |
| IL-10 | 11.818 | <0.001 | 0.665 | 0.418 | 1.488 | 0.230 |
| MIP-3α | 5.304 | 0.006 | 0.825 | 0.367 | 1.383 | 0.255 |
| IL-12P70 | 7.553 | 0.001 | 0.421 | 0.519 | 1.514 | 0.224 |
| IL-13 | 1.320 | 0.271 | 0.562 | 0.456 | 0.882 | 0.416 |
| IL-17α | 9.649 | <0.001 | 0.804 | 0.373 | 2.024 | 0.136 |
| IL-1β | 5.742 | 0.004 | 1.976 | 0.164 | 6.876 | 0.001 |
| IL-2 | 11.669 | <0.001 | 0.463 | 0.499 | 1.300 | 0.276 |
| IL-4 | 3.759 | 0.026 | 1.488 | 0.227 | 1.937 | 0.148 |
| IL-23 | 12.541 | <0.001 | 1.960 | 0.166 | 1.115 | 0.331 |
| IL-5 | 4.893 | 0.009 | 0.072 | 0.789 | 2.482 | 0.087 |
| IL-6 | 3.710 | 0.027 | 2.175 | 0.145 | 3.174 | 0.045 |
| IL-7 | 5.489 | 0.005 | 2.324 | 0.132 | 3.459 | 0.054 |
| IL-8 | 5.532 | 0.005 | 0.055 | 0.815 | 0.007 | 0.993 |
| MIP-1β | 4.929 | 0.009 | 0.002 | 0.968 | 1.353 | 0.262 |
| TNF-α | 14.515 | <0.001 | 1.859 | 0.177 | 0.707 | 0.495 |

Abbreviations: ITAC, interferon-inducible T cell alpha chemoattractant; GM-CSF, granulocyte macrophage colony-stimulating factor; IFN, interferon; IL, interleukin; MIP, macrophage inflammatory protein; TNF, tumor necrosis factor.
